# Inferior vena cava distensibility from subcostal and trans-hepatic imaging using both M-mode or artificial intelligence: a prospective study on mechanically ventilated patients

**DOI:** 10.1186/s40635-023-00529-z

**Published:** 2023-07-10

**Authors:** Filippo Sanfilippo, Luigi La Via, Veronica Dezio, Paolo Amelio, Giulio Genoese, Federico Franchi, Antonio Messina, Chiara Robba, Alberto Noto

**Affiliations:** 1 Department of Anaesthesia and Intensive Care, A.O.U. Policlinico-San Marco, Site “Policlinico G. Rodolico”, Via S. Sofia N 78, 95123 Catania, Italy; 2 School of Anaesthesia and Intensive Care, University Hospital “G. Rodolico”, University of Catania, 95123 Catania, Italy; 3 School of Anaesthesia and Intensive Care, University “Magna Graecia”, Catanzaro, Italy; 4 Division of Anesthesia and Intensive Care, University of Messina, Policlinico “G. Martino”, Messina, Italy; 5 Anesthesia and Intensive Care Unit, University Hospital of Siena, University of Siena, Siena, Italy; 6 Humanitas Clinical and Research Center, IRCCS, Milan, Italy; 7 Department of Biomedical Sciences, Humanitas University, Pieve Emanuele, MI Italy; 8 Department of Surgical Science and Diagnostic Integrated, University of Genoa, Genoa, Italy; 9 IRCCS Ospedale Policlinico San Martino, Genoa, Italy; 10 Department of Human Pathology of the Adult and Evolutive Age “Gaetano Barresi”, Division of Anesthesia and Intensive Care, University of Messina, Policlinico “G. Martino”, Messina, Italy

**Keywords:** Critical care, Ultrasound, Subcostal, Transhepatic, Inferior vena cava

## Abstract

**Background:**

Variation of inferior vena cava (IVC) is used to predict fluid-responsiveness, but the IVC visualization with standard sagittal approach (SC, subcostal) cannot be always achieved. In such cases, coronal trans-hepatic (TH) window may offer an alternative, but the interchangeability of IVC measurements in SC and TH is not fully established. Furthermore, artificial intelligence (AI) with automated border detection may be of clinical value but it needs validation.

**Methods:**

Prospective observational validation study in mechanically ventilated patients with pressure-controlled mode. Primary outcome was the IVC distensibility (IVC-DI) in SC and TH imaging, with measurements taken both in M-Mode or with AI software. We calculated mean bias, limits of agreement (LoA), and intra-class correlation (ICC) coefficient.

**Results:**

Thirty-three patients were included. Feasibility rate was 87.9% and 81.8% for SC and TH visualization, respectively. Comparing imaging from the same anatomical site acquired with different modalities (M-Mode vs AI), we found the following IVC-DI differences: (1) SC: mean bias − 3.1%, LoA [− 20.1; 13.9], ICC = 0.65; (2) TH: mean bias − 2.0%, LoA [− 19.3; 15.4], ICC = 0.65. When comparing the results obtained from the same modality but from different sites (SC vs TH), IVC-DI differences were: (3) M-Mode: mean bias 1.1%, LoA [− 6.9; 9.1], ICC = 0.54; (4) AI: mean bias 2.0%, LoA [− 25.7; 29.7], ICC = 0.32.

**Conclusions:**

In patients mechanically ventilated, AI software shows good accuracy (modest overestimation) and moderate correlation as compared to M-mode assessment of IVC-DI, both for SC and TH windows. However, precision seems suboptimal with wide LoA. The comparison of M-Mode or AI between different sites yields similar results but with weaker correlation.

*Trial registration* Reference protocol: 53/2022/PO, approved on 21/03/2022

**Supplementary Information:**

The online version contains supplementary material available at 10.1186/s40635-023-00529-z.

## Introduction

Evaluation of fluid responsiveness (FR) has a prominent role in the treatment of intensive care unit (ICU) patients; in fact, both fluid overload or dehydration have been demonstrated a negative impact on morbidity and mortality of critically ill patients [1]. Hypovolemia and reduced preload are responsible for a reduction in stroke volume, thus causing organ hypo-perfusion [2], while hypervolemia impairs organ perfusion by determining fluid overload, with consequent tissue edema and pulmonary and/or systemic congestion [3–5]. Therefore, ICU patients usually require the evaluation of FR several times a day [6], since loading conditions tend to be modified by different variables (vasomotor tone [7], analgo-sedation level, capillary permeability related to inflammation [8], etc.).

Prediction of FR can be performed with several methods, both in spontaneously breathing and mechanically ventilated patients [9, 10]. Some are non-invasive or minimally invasive, while others require advanced cardiac output monitoring and arterial cannulation. Among the non-invasive ones, variation of inferior vena cava (IVC) diameter within the respiratory cycle is commonly adopted, and it has been validated for both mechanically ventilated patients (IVC distensibility, IVC-DI = ΔIVC/IVCmin, with 18% as best cutoff) [11], and for patients with spontaneous respiratory activity (IVC collapsibility, IVCc = ΔIVC/IVCmax, with cutoffs around 40–48%) [12–14]. The IVC assessment is highly feasible at the bedspace, thus explaining the growing application in most critically ill patients [15, 16]; however, it must be acknowledged that there are several limitations in the use of IVC for the reliable prediction of FR [17–19]. Moreover, standard subcostal (SC or sagittal) approach for the IVC assessment is not always achievable as it happens in case of laparotomy wounds, presence of chest drains, obesity or enlarged bowel. In these instances, the trans-hepatic (TH, coronal, or right lateral) approach for IVC visualization could be an alternative, offering a latero-lateral visualization of the vessel excursions. Available data on the interchangeability of IVC assessments with SC and TH approach are conflicting [20, 21], and a systematic review showed limited evidence to draw conclusions. Indeed, the available studies are grossly heterogeneous and used different approaches in data reporting, suggesting the need for further research [22].

In the past decade the role of artificial intelligence (AI) grew rapidly in several medical fields. Among these, also echocardiography is experiencing a significant expansion of AI applications that might help daily practice. Indeed, AI has been used for the assessment of left ventricular systolic [23, 24] and diastolic [25–27] function, right ventricular function [28], but also for the evaluation of heart valve [29] and congenital heart diseases [30]. Moreover, machine learning has been developed for predicting FR at patient’s bedside [31] with preliminary data on the implementation of AI for IVC assessment [32].

We conducted a prospective observational study in mechanically ventilated critically ill patients to compare differences in IVC size and variation between measurements taken in traditional M-Mode or with AI approach, as well as to evaluate the differences between measurements taken at the two different anatomical sites (SC and TH).

## Materials and methods

Our prospective observational study was approved from our local Ethical Committee (Reference protocol: 53/2022/PO) before enrolling the first patient (21/03/2022). We aimed at evaluating the differences between assessment of the IVC in SC and TH windows. We previously conducted a proof-of concept study with the same approach on healthy volunteers [33].

### Participants

We included adult patients admitted to the General ICU of the *Azienda Ospedaliera Universitaria “Policlinico-San Marco”, Catania* if they were fully ventilated in pressure-controlled ventilation (PCV) without own respiratory activity and stable hemodynamic conditions and if the operator (FS) was available on shift. We collected data as suggested by the PRICES guidelines [34, 35], recording ICU admission diagnosis, patient’s demographics, the hemodynamic conditions and the ventilatory settings, and ICU mortality.

### Study procedure

All patients were in semi-recumbent (35°) position. An experienced certified operator (FS) acquired IVC four types of imaging (SC or TH view, standard M-mode or with the aid of AI, see Additional file 1), using the same portable ultrasound machine *General Electric (GE) Venue Go R2*. The AI images were acquired with the “*auto IVC tool*” available exclusively on the GE Healthcare Venue family, which automates the IVC assessment to accurately deliver the IVC diameter and collapsibility/distensibility immediately [36]. The operator attempted acquire the images as close as possible to the cavo-atrial junction and not farer than 4 cm. Moreover, the operator tried to minimize any cranio-caudal IVC displacements during the respiratory cycle, measuring the diameters at the same distance from the cavo-atrial junction.

### Offline calculation procedure

Images were stored in the ultrasound machine and downloaded separately. The calculation of the IVC diameters and of the IVC-DI was performed subsequently offline. In case of the IVC size analysis in M-mode, we analyzed a single measure which was the most reliable one as decided by the experienced operator performing the offline calculation.

The AI imaging were gathered with automated contour tracking function for the detection of IVC borders; each clip lasted 6 s. In case of the AI, repeated images were acquired and saved in the database. The images and the relative automated data on the diameters and IVC-DI were subsequently reviewed offline checking for artifacts and errors. When reviewing the AI images and the data, the operators were blinded from the M-mode data.

### Study groups and outcomes

Four groups of data were generated from the combination of the view of image acquisition (SC or TH) and the data calculation modality: (1) SC in M-mode; (2) SC in AI; (3) TH in M-mode; (4) TH in AI. Our study had a factorial 2 × 2 design, comparing the differences and correlations of IVC measurements according to:

A. *Different measuring modality****:*** the same site of acquisition but with different acquisition modality (M-mode vs AI), thus comparing:
  SC-in M-mode vs SC in AI; and
  TH in M-mode vs TH in AI;
B. *Different acquisition view:* the same measuring modality with different view of imaging (SC vs TH), thus comparing:
  SC in M-mode vs TH in M-mode; and
  SC in AI vs TH in AI.

The variable of primary interest in our study was the IVC-DI. As secondary endpoints we analyzed the IVC diameters (IVCmax and IVCmin).

### Statistical analysis

A study reported high correlation between SC and TH imaging of the IVC (Pearson coefficient *r* = 0.86), but the authors included a heterogeneous population of patients ventilated in pressure support or PCV, as well as patients on non-invasive ventilation and high-flow nasal oxygen [37]. Conversely, another study reported much lower correlation coefficients (0.14–0.32) [38]. The impression from a systematic review conducted on this argument [22] and including seven studies was that overall agreement between the two approaches is moderate at best. Therefore, based on agreement between authors our sample size was calculated assuming a statistical power of 80% and an α level at 0.05, with a correlation coefficient estimated at *r* = 0.55. The resulted sample size calculation was *n* = 24. Due to paucity of data regarding the likely correlation between M-Mode and AI data, we did not formally calculate a sample size for the arm of the study focusing on comparison of M-Mode and AI data.

We calculated the agreements mean bias, and limits of agreement [LOA] between IVC measurements in different areas/modalities with the Bland and Altman plots. Bland–Altman plots and statistics were adjusted for the effect of multiple measures as described by Zou only for the comparison of AI modalities [39]. The bias indicates the accuracy of measurements methods, while the LOA specifies the precision. Their values are reported with the relative 95% confidence interval. Considering that the best cutoff for prediction of FR using the IVC-DI in mechanically ventilated patients is considered 18%, we decided that a mean bias of 4% and 2% would describe acceptable and good accuracy, respectively. Regarding the precision (LOA) of the measurements, we considered a range of 16% and 8% as acceptable and good precision, respectively. The relationship among variables was evaluated calculating the intra-class correlation (ICC) coefficient to describe the inter-rater variability between measures acquired with the same modality (AI TH vs AI SC, or M-mode TH vs M-mode SC) or in the same approach (AI TH vs M-mode TH, or AI SC vs M-mode SC) resemble each other. Interpretation of correlation was performed according to established cutoffs [40].

From clinical perspectives, considering that a value above 18% for IVC-DI gathered in M-mode SC is the commonly adopted cutoff for FR, we report the number of cases, where M-mode TH was in agreement or not with M-mode SC assessment in respect to FR.

## Results

Descriptive statistics of the patients participating in the study are reported in Table 1. Main diagnosis of admission and severity scores are provided separately as Additional file 2. Of the 33 patients included, one did not have any acoustic window (3%) and was excluded; further three patients did not have SC view (9.1%) and for other five (15.2%) it was not possible to obtain the TH visualization. Overall feasibility was 87.9% for SC imaging and 81.8% for TH visualization. The mean IVC-DIs were 14.8 ± 7.9% and 15.1 ± 8.5% for SC and TH imaging, respectively.

**Table 1:** Characteristics of the study population and average results of the inferior vena cava (IVC) distensibility, minimum and maximum diameters (IVC-DI, IVC-min and IVC-max, respectively) calculated in subcostal (SC) and transhepatic (TH) windows

Baseline characteristics and measurements		Ventilatory settings and Hemodynamics	
Gender (male)	25/32 (78%)	PEEP (cmH_2_O)	6 ± 1
Age (years)	65 ± 13	Pressure Control (cmH_2_O)	16 ± 6
Weight (Kg)	80 ± 21	Tidal Volume (ml)	517 ± 124
Height (cm)	170 ± 7	Respiratory Rate (bpm)	16 ± 3
		SaO_2_ (%)	98 ± 3
IVCmin in SC (mm, M-mode)	20.8 ± 4.4	Heart rate (bpm)	83 ± 19
IVCmax in SC (mm, M-mode)	23.7 ± 4.3	Sinus rhythm (*n* =)	29/32
IVC-DI in SC (%, M-mode)	14.8 ± 7.9	SAP (mmHg)	106 ± 22
IVCmin in TH (mm, M-mode)	19.8 ± 4.2	MAP (mmHg)	74 ± 14
IVCmax in TH (mm, M-mode)	22.6 ± 4.2	DAP (mmHg)	58 ± 12
IVC-DI in TH (%, M-mode)	15.1 ± 8.5	PPV (%)	13 ± 10
		Norepinephrine (mcg/kg/min)	0.33 ± 0.22
SOFA score	12.2 ± 3.7	Vasoactive (*n* =)	20/32 (63%)
Mortality	22/32 (69%)	Second vasoactive drug (*n* =)	5 (16%)

*DAP* diastolic arterial pressure, *MAP* mean arterial pressure, *PPV* pulse pressure variation, *PEEP* positive end-expiratory pressure, *SAP* systolic arterial pressure. Data are reported as mean and standard deviation

Results of the Bland Altman plots are reported in Table 2, where the mean bias, the lower and the upper LOA with their 95%CI are shown. In the same table, we report also the Spearman rho and ICC to describe how strong measurements resemble each other.

**Table 2:** Summary of comparisons between measurement of the inferior vena cava (IVC) in adult patients mechanically ventilated in pressure control mode

Comparison		Variable	ICC 95%CI	Mean bias 95%CI	Lower LOA 95% CI	Upper LOA 95% CI
M-SC	AI-SC	IVC Min (mm)	0.79; − 0.01 to 0.93	3.0; 2.0 to 4.0	− 2.1; − 3.8 to − 0.3	8.1; 6.3 to 9.8
		IVC Max (mm)	0.78; 0.02 to 0.93	2.9; 1.9 to 3.9	− 2.3; − 4.1 to − 0.5	8.1; 6.4 to 9.9
		**IVC-DI (%)**	**0.65;** **0.27 to 0.83**	**− 3.1;** **− 6.4 to 0.3**	**− 20.1;** **− 25.9 to − 14.3**	**13.9;** **8.1 to 19.7**
M-TH	AI-TH	IVC Min (mm)	0.88; − 0.06 to 0.96	2.4; 1.8 to 3.1	− 0.7; − 1.8 to 0.4	5.6; 4.5 to 6.7
		IVC Max (mm)	0.85; − 0.08 to 0.96	2.5; 1.9 to 3.2	− 0.8; − 2.0 to 0.3	5.9; 4.7 to 7.1
		**IVC-DI (%)**	**0.65;** **0.25 to 0.84**	**− 2.0;** **− 5.5 to 1.5**	**− 19.3;** **− 25.4 to 13.3**	**15.4;** **9.3 to 21.5**
M-SC	M-TH	IVC Min (mm)	0.74; 0.41 to 0.88	1.1; − 0.7 to 2.8	− 6.9; − 9.9 to − 4.0	9.1; 6.1 to 12.0
		IVC Max (mm)	0.69; 0.30 to 0.86	1.2; − 0.5 to 3.0	− 7.0; − 10.0 to -3.9	9.5; 6.4 to 12.5
		**IVC-DI (%)**	**0.54;** **− 0.09 to 0.80**	**0.1;** **− 4.0 to 4.2**	**− 19.0;** **− 26.2 to − 11.9**	**19.3;** **12.1 to 26.4**
AI-SC	AI-TH	IVC Min (mm)	0.77; 0.46 to 0.90	0.4	− 6.9; − 9.9 to − 4.9	7.8; 5.75 to 10.82
		IVC Max (mm)	0.76; 0.45 to 0.90	0.9	− 6.0; − 8.9 to − 4.1	7.8; 5.9 to 10.7
		**IVC-DI (%)**	**0.32;** **− 0.63 to 0.72**	**2.0**	**− 25.7;** **− 35.4 to − 19.3**	**29.7;** **23.3 to 39.4**

In case of the IVC size analysis in M-mode (M), we analyzed a single measure which was the most reliable measure as decided by the experienced operator performing the calculations. In case of the analysis with artificial intelligence (AI), repeated measures were taken and saved in the database. Results of IVC distensibility, minimum and maximum diameters (IVC-DI, IVC-min and IVC-max, respectively) are provided in terms of mean Bias and limits of agreement (LoA) with their relative 95% confidence interval (CI) where appropriate. We also provide intraclass correlation coefficient (ICC) to describe how strong the measurements resemble each other

The IVC-DI was the primary outcome and it is indicated in bold font

### Different acquisition modality

**Fig. 1:**
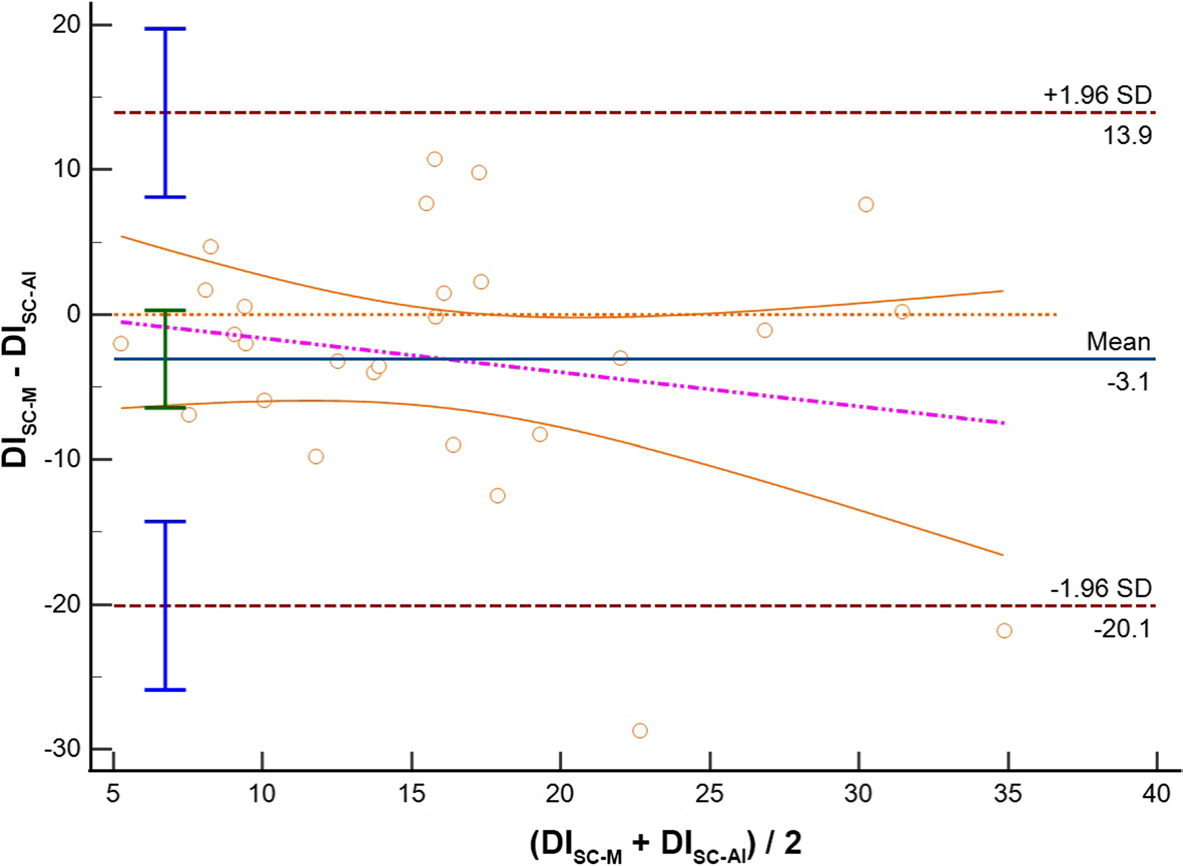
Bland–Altman plot for the inferior vena cava distensibility index (DI) measured in subcostal site with standard M-Mode (SC-M) or artificial intelligence (SC-AI). *SD* standard deviation

**Fig. 2:**
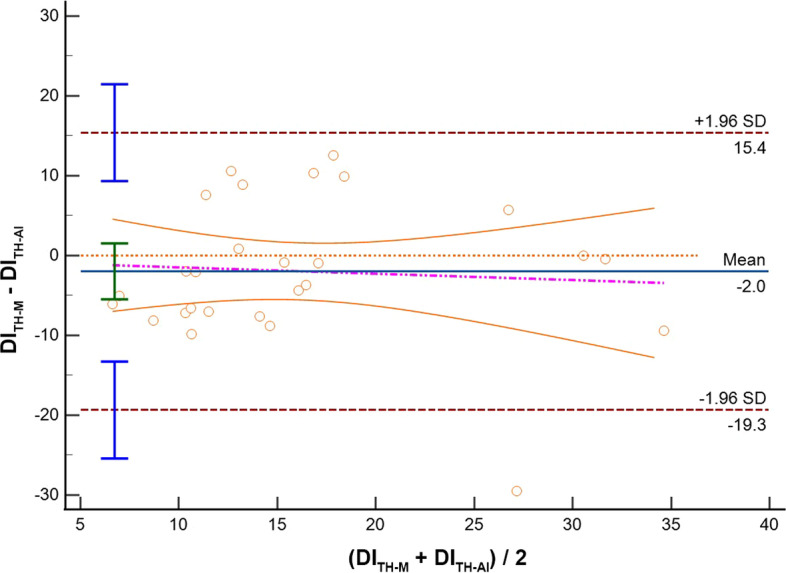
Bland–Altman plot for the inferior vena cava distensibility index (DI) measured in Transhepatic site with standard M-Mode (TH-M) or artificial intelligence (TH-AI). *SD* standard deviation

Comparing M-mode and AI strategy for IVC assessment, measurements, where similar both for SC IVC-DI (bias − 3.1%, LoA [− 20.1; 13.9], Fig. 1) and diameters (IVCmax: bias 2.9 mm, LoA [− 2.3; 8.1]; IVCmin: bias 3.0 mm, LoA [− 2.1; 8.1]), as well as for the TH IVC-DI (bias − 2.0%, LoA [− 19.3; 15.4]; Fig. 2) and diameters (IVCmax: bias 2.5 mm, LoA [− 0.8; 5.9]; IVCmin: bias 2.4 mm, LoA [− 0.7, 5.6]). As shown by the violet dotted line in the Bland–Altman plots of Fig. 1, there was a clear trend in the bias for the IVC-DI when calculated in SC imaging: precisely, a lower bias between M-Mode and AI was seen when the IVC-DI was approaching 5%. Conversely, a trend in the bias between modalities of IVC-DI calculation was not present in the case of TH imaging.

Overall, the ICC coefficients showed moderate-to-good reliability; in particular, the ICC of the IVC-DI was 0.65 [0.25, 0.84] for SC imaging, and 0.65 [0.27, 0.83] for the TH window.

### Different acquisition site

**Fig. 3:**
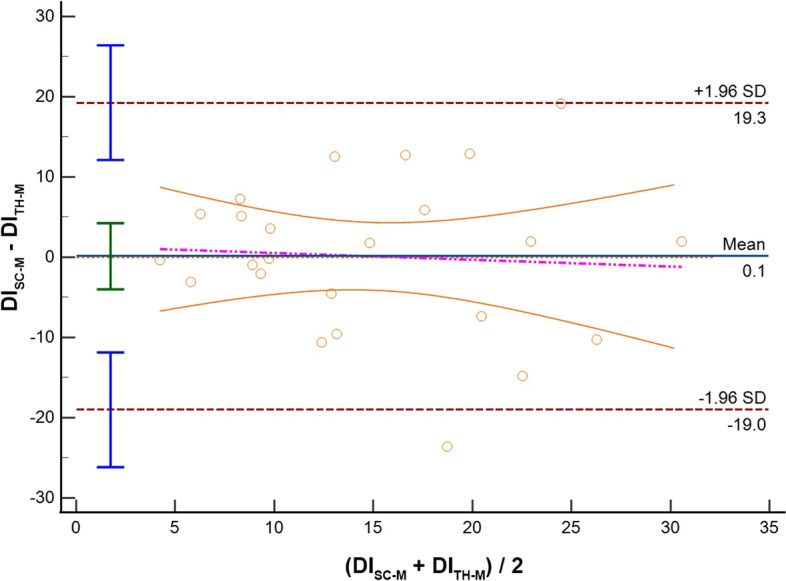
Bland–Altman plot for the inferior vena cava distensibility index (DI) measured with standard M-Mode in two different sites: subcostal (SC-M) and transhepatic (TH-M). *SD* standard deviation

**Fig. 4:**
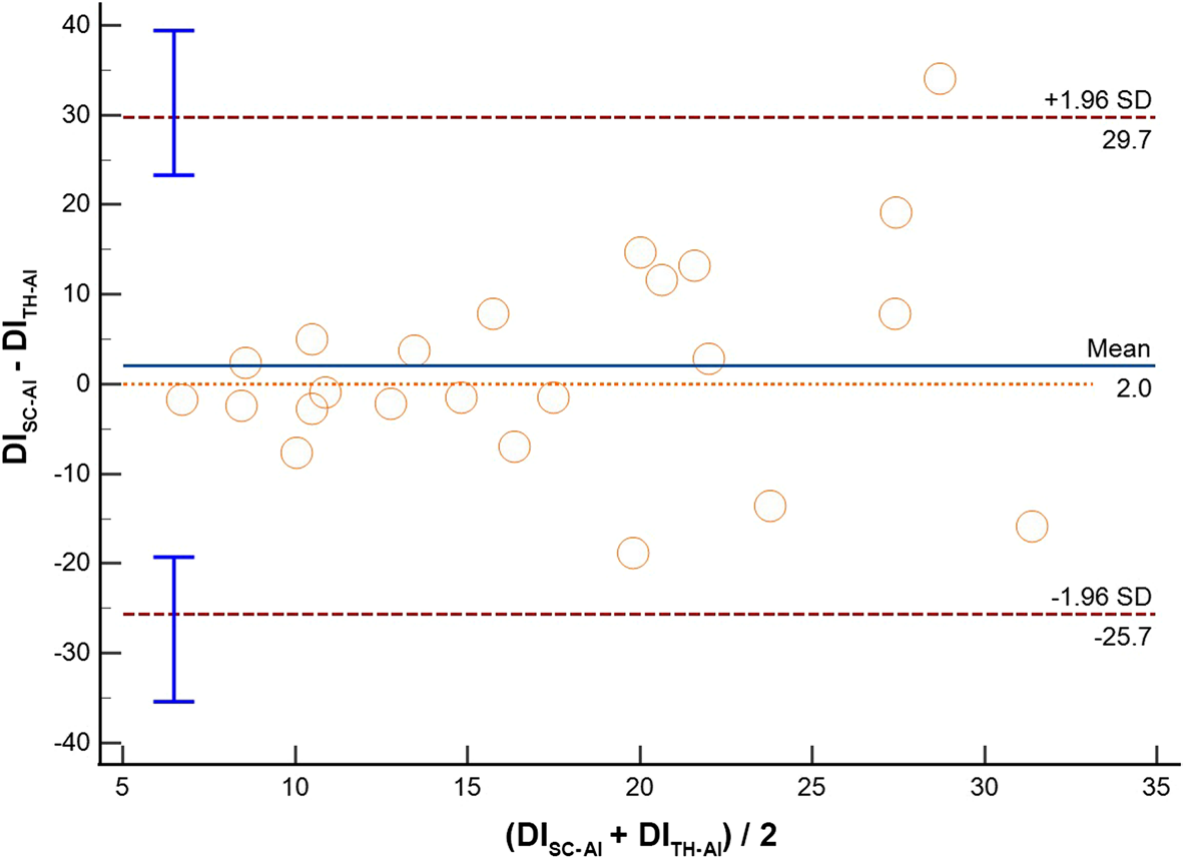
Bland–Altman plot for the inferior vena cava distensibility index (DI) measured with artificial intelligence mode in two different sites: subcostal (SC-AI) and transhepatic (TH-AI). *SD* standard deviation

When the assessments of the IVC were compared between anatomical sites (SC vs TH) we found that comparing the SC and TH M-mode assessment, IVC-DI had a mean bias 0.1% with LoA [− 19.0; 19.3] (Fig. 3); also, the IVC diameters showed differences between anatomical sites (IVCmax: bias 1.2 mm, LoA [− 7.0; 9.5]; IVCmin: bias 1.1 mm, LoA [− 6.9; 9.1]). When the evaluation was performed with the aid of AI, the differences between SC and TH seemed slightly higher for the IVC-DI (bias 2.0%, LoA [− 25.7; 29.7]; Fig. 4) and the diameters (IVCmax: bias 0.9 mm, LoA [− 6.0; 7.8]; IVCmin: bias 0.4 mm, LoA [− 6.9, 7.8]). The correlation of IVC-DI seemed slightly weaker when comparing the SC and the TH windows, with ICC ranging between 0.32 and 0.54 (Table 2).

### Agreement in respect to FR

Among the 24 couples of patients with M-mode view obtained from both SC and TH approach, we found that in 16 cases (67%) the assessment with M-mode TH was concordant with the indication on FR gathered from M-mode SC (*n* = 13 both below the cutoff, *n* = 3 both above the cutoff). In the remaining 8 cases (33%), the M-mode TH indicated FR not suggested by M-mode SC (*n* = 3) or conversely did not confirm a FR suggested by M-mode SC (*n* = 5).

## Discussion

Our study evaluated the assessment of the IVC-DI (and of the IVC diameters) at two different anatomical regions, comparing results obtained from a standard sagittal view (SC approach) with those gathered with coronal approach (TH). Of note, our study not only evaluated the accuracy of the standard method (M-Mode), but also introduced the evaluation of the IVC-DI and diameters results acquired with an automated modality (AI). The main finding of our study conducted with multiple comparisons was an overall acceptable/good accuracy between both acquisition modality (M-mode vs AI) or site (SC vs TH) with mean bias ranging between − 3.1% and + 2%, but we found suboptimal precision as demonstrated by relatively wide upper and lower LoA. Such lack of precision reduces the clinical utility of the alternative approaches (TH or AI), as a significant number of patients could be classified as fluid responders by one method and as non-responders by another one. As the present investigation has features of a 2 × 2 factorial study linking evaluations of the IVC from different technical and anatomical standpoints, we separate the discussion of our results in two parts. First, we discuss results focused on the validation of AI measurements for IVC assessment, and thereafter debate the differences between imaging in SC (sagittal) or TH (coronal) approach.

### Comments on results from different acquisition modality

As other studies evaluated the differences between SC and TH imaging in mechanically ventilated patients [20, 37, 41], we focus first on the differences between M-Mode and AI measurements, with particular emphasis on the results on the IVC-DI, which is the one used by clinicians for decision-making at the bed-space. Our results suggest that introduction of AI could have some clinical value; indeed, we found that accuracy of AI calculation was between acceptable and good according to predefined interpretation cutoffs. In particular, AI overestimate the IVC-DI both for TH and SC approach (mean bias − 2% for TH and of − 3.1% for SC). However, in both cases we found suboptimal precision comparing the M-Mode and the AI measurements as demonstrated by relatively wide upper and lower LoA, with roughly a 17% difference from the mean bias. In this context, it must be considered that estimating the diameters when the IVC is almost fully collapsible (i.e., IVCmin below 0.5 mm) is technically challenging. In such cases, the evaluation in M-Mode using the touch screen (as for the ultrasound machine in our study) may be prone to smaller mistakes that could affect precision of the measurements, finally influencing the LOA. Although the accuracy of TH imaging (− 2%) may seem greater than SC (− 3.1%), it is important to note from a clinical perspective that the Bland–Altman plot of the SC imaging (Fig. 1) showed a clear trend bias for the IVC-DI (violet dotted line). In particular, as compared to AI calculation, the M-Mode seems underestimating the IVC-DI, with greater differences between modalities seen for the higher values of IVC-DI (i.e., fluid responders). Indeed, we noted that mean bias approaches the “zero” value (excellent accuracy between methods) when the IVC has limited excursion with minor changes in its diameter during respiration (IVC-DI close to 5%). Summarizing, it seems that AI offers an accurate reproduction of M-Mode calculations for the IVC-DI; thus, AI introduction for automated border detection may be great assistance for clinicians in daily practice, with potentialities of saving time for bedside assessment of volume status. Moreover, the use of AI may allow a larger number of IVC-DI calculations that could be averaged, with possible advantages in cases of borderline IVC-DI results. From practical perspectives, instead of freezing the ultrasound image, to measure the IVC diameters and to apply the IVC-DI formula, with the help of AI the sonographer/physician can just hold the probe focused on the IVC, while the ultrasound machine calculates values of IVC-DI (or eventually IVC collapsibility index according to the type of ventilation selected). The use of AI has been applied to the whole echocardiography setting (i.e., left ventricular systolic [23, 24] and diastolic [25–27] function, to right ventricular function [28], assessment of heart valve diseases [29], diagnosis of congenital heart diseases [30]) and also to predict of FR, with encouraging results. For instance, Bataille et al. [31] showed that machine learning models predicted FR with comparable accuracy to the hemodynamic response to passive leg raising, and evaluation of the IVC was among the key variables identified by the model, together with other Doppler derived parameters. Blaivas et al. used a deep learning algorithm capable of video classification for the estimation of FR using IVC imaging, and demonstrated that the trained algorithm had moderate performances with an area under the curve of 0.70 (95%CI 0.43–1.00) [32]. Furthermore, the same group verified that the performances of this algorithm were dependent on the quality of the IVC image with significantly worse performances on images of lower quality [42]. The findings of our validation study pooled together with the other few studies available suggests that it could be worth to introduce AI with automated contour tracking of IVC in daily clinical practice, with good accuracy of the AI as compared to the M-Mode, although the precision of the method may be suboptimal. However, in our study we did not assess FR; consequently, our comparison between M-mode and AI modality for IVC-DI cannot focus on diagnostic performances but rather on interchangeability of measurements at individual level.

### Comments on results from different acquisition site

Our study investigated also the interchangeability of IVC-DI recorded in SC and in TH approaches. A recent systematic review included seven studies, suggested that results of SC and TH imaging for IVC-DI may be not fully interchangeable. However, the evidence comes from a very heterogeneous cohorts of participants, being present studies on both volunteers, spontaneously breathing and/or mechanically ventilated patients [22]. We observed small mean biases of IVC-DI between SC and TH imaging obtained both in case of M-Mode or AI measurements (0.1% and 2%, respectively), and this finding is not novel, as similar ones have been reported for M-Mode measurements by two studies (IVC-DI mean bias of 0.5% [20] and − 0.5% [37]), while to the best of our knowledge, no study has compared SC and TH with the aid of AI. Despite the small mean biases, we confirmed wide LoA for both M-Mode and AI methods (roughly 19% and 28%, respectively), suggesting suboptimal precision and partially discouraging the interchangeability of measurements between sites. Interestingly, the use of AI did not improve the accuracy or precision as compared to the M-Mode calculations.

Nonetheless, regardless the interchangeability of SC and TH imaging, we believe that TH window is easy even in novice hands and can be clinically useful, especially when the sagittal (SC) imaging cannot be achieved (i.e., obesity, for the presence of laparotomy wound, mediastinal drains, etc.). Thus, research should be encouraged for the investigation of cutoffs for predicting FR using the IVC in coronal view (TH). In this context, the feasibility of TH imaging in our study was 82%, very similar to the one reported by Valette et al. (81%) [37].

### Strengths and limitations of the study

The main strengths of this study regard the use of AI for both validating this method as compared to the reference method (M-Mode) and for investigating the differences between SC and TH imaging. We conducted a study in a homogeneous population of mechanically ventilated patients with PCV mode with an average of 8 ml/kg of tidal volume and a low positive end expiratory pressure (5.9 cmH_2_O). Most of the patients recruited was on vasopressor support (average norepinephrine 0.32 mcg/kg/min) and with a pulse pressure variation on the edge of FR (13%), resembling a typical population, where assessment of FR may be clinically needed. Overall, our study seemed adequately powered as we recruited 24 patients with both SC and TH imaging as per sample size estimation, where we assumed a correlation coefficient of 0.55. Of note, as compared to this assumed value, we found a higher ICC in the comparison between methods (M-Mode vs AI) in the same anatomical area (*r* = 0.65 for both the SC and the TH areas), and a similar ICC when comparing different areas with M-Mode approach (*r* = 0.54). Only the ICC evaluating different areas with AI approach resulted much lower (*r* = 0.32), and therefore, only the data on AI may be underpowered.

Our study has also several limitations. First, our cohort was smaller than the other two studies on ventilated patients [20, 41] with fully controlled mechanical ventilation. Second, a single experienced operator collected the images and performed M-mode calculations, and results may be different in less experienced hands. Moreover, the study was performed with a single vendor's AI. Other vendors may have subtle differences in their tracking technology, thus resulting in different accuracy and/or precision, as it happens for speckle tracking technology. Third, we did not perform fluid challenge in our population, so that we cannot calculate cutoffs and area under the curve for the prediction of FR. Fourth, the image acquisition followed a schematic pattern starting from SC imaging and moving to TH approach after to avoid human mistakes in data collection, but an ideal methodological design would have provided randomization of the order of image recording. Nonetheless, we believe this is unlikely to influence results but it remains fair to acknowledge such item.

## Conclusions

The use of artificial intelligence for the evaluation of the inferior vena cava distensibility index seem to have good accuracy when compared with standard M-mode assessment, both in case of subcostal or transhepatic imaging. However, the precision of the method is suboptimal. Artificial intelligence does not seem to reduce the differences in inferior vena cava distensibility between SC and TH imaging, and results from these two anatomical sites yield low precision.

## Supplementary Information

**Additional file 1.** An example of inferior vena cavaimages taken from subcostal or transhepatic view. On the left side of the figure, images are acquired from subcostal window in M-modeor with aid of artificial intelligence. Similarly, on the right images are obtained from transhepatic window with M-mode at the top and AI at the bottom corner. In the AI images, automated calculation of the distensibility indexis shown.

**Additional file 2.** Main diagnosis of admission for patients included and their severity score.

## Data Availability

Data available from corresponding author at reasonable request.

## Abbreviations

FR: Fluid responsiveness
ICU: Intensive care unit
IVC: Inferior vena cava
IVC-DI: IVC distensibility
IVCc: IVC collapsibility
SC: Subcostal
TH: Trans-hepatic
AI: Artificial intelligence
LOA: Limits of agreement
ICC: Intra-class correlation
