# Supplementary figures and images for: Inferior vena cava distensibility from subcostal and trans-hepatic imaging using both M-mode or artificial intelligence: a prospective study on mechanically ventilated patients
Source: Intensive Care Med Exp. 2023 Jul 10;11:40. doi: 10.1186/s40635-023-00529-z (PMC10329966; doi:10.1186/s40635-023-00529-z)

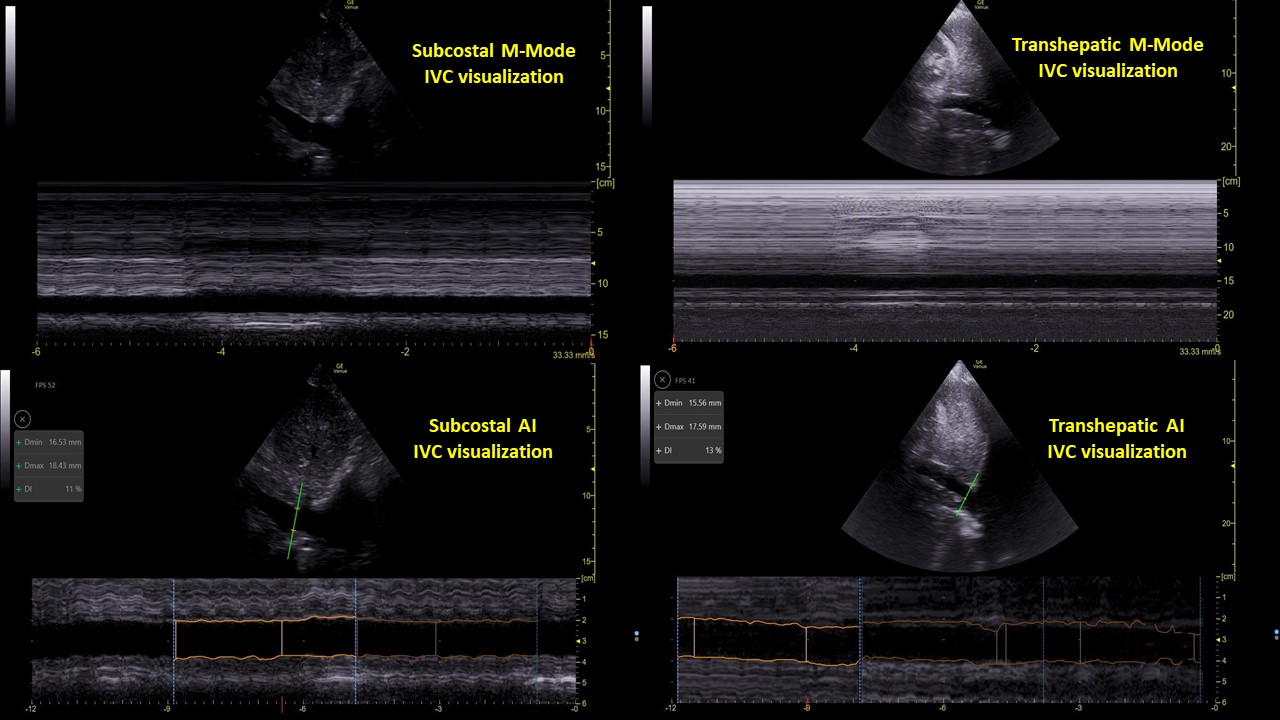

Supplement: Supplementary file 1 — Additional file 1. An example of inferior vena cavaimages taken from subcostal or transhepatic view. On the left side of the figure, images are acquired from subcostal window in M-modeor with aid of artificial intelligence. Similarly, on the right images are obtained from transhepatic window with M-mode at the top and AI at the bottom corner. In the AI images, automated calculation of the distensibility indexis shown. [file 40635_2023_529_MOESM1_ESM.jpg]
