# Supplementary material for: Inferior vena cava distensibility from subcostal and trans-hepatic imaging using both M-mode or artificial intelligence: a prospective study on mechanically ventilated patients
Source: Intensive Care Med Exp. 2023 Jul 10;11:40. doi: 10.1186/s40635-023-00529-z (PMC10329966; doi:10.1186/s40635-023-00529-z)
Supplement: Supplementary file 2 — Additional file 2. Main diagnosis of admission for patients included and their severity score. [file 40635_2023_529_MOESM2_ESM.docx]

**Additional material 1**

1. Main diagnosis of admission and severity score (SOFA Score)

| **n** | Diagnosis of admission | **Outcome** | **SOFA Score** |
| --- | --- | --- | --- |
|  | ROSC | **ICU mortality** | **15** |
|  | Acute on Chronic Respiratory Failure | **Transfer** | **10** |
|  | Ruptured Ascending Aortic Aneurysm | **Transfer** | **14** |
|  | Politrauma | **Transfer** | **7** |
|  | Hematological GVHD | **ICU mortality** | **14** |
|  | OHCA - ROSC | **ICU mortality** | **11** |
|  | ARDS | **ICU mortality** | **16** |
|  | Septic shock | **ICU mortality** | **9** |
|  | Acute Respiratory Failure | **ICU mortality** | **18** |
|  | Acute Respiratory Failure | **ICU mortality** | **15** |
|  | Acute Respiratory Failure | **ICU mortality** | **18** |
|  | Acute Respiratory Failure in Lymphoma | **Trasnsfer** | **14** |
|  | Guillan-Barre Syndrome | **ICU mortality** | **13** |
|  | ROSC | **ICU mortality** | **14** |
|  | ROSC | **ICU mortality** | **13** |
|  | ROSC | **Transfer** | **9** |
|  | ARDS | **Transfer** | **7** |
|  | Convulsive Epileptic Disorder | **Transfer** | **10** |
|  | Acute Respiratory Failure | **ICU mortality** | **18** |
|  | Acute Respiratory Failure and Hydrocephalus | **ICU mortality** | **10** |
|  | Endocarditis and Septic Shock | **ICU mortality** | **18** |
|  | Acute on Chronic Respiratory Failure | **ICU mortality** | **15** |
|  | ROSC | **ICU mortality** | **13** |
|  | Intracerebral Haemorrhage | **ICU mortality** | **10** |
|  | Intracerebral Haemorrhage | **ICU mortality** | **6** |
|  | Decompensated Heart Failure | **ICU mortality** | **14** |
|  | Intracerebral Haemorrhage | **Transfer** | **10** |
|  | ROSC | **Transfer** | **14** |
|  | Acute Respiratory Failure | **ICU mortality** | **5** |
|  | Traumatic Brain Injury | **Transfer** | **9** |
|  | Acute on Chronic Respiratory Failure | **ICU mortality** | **15** |
|  | Acute on Chronic Respiratory Failure | **ICU mortality** | **12** |
|  | ROSC | **Transfer** | **7** |

Supplementary
